# Occurrence of Plasmids in the Aromatic Degrading Bacterioplankton of the Baltic Sea

**DOI:** 10.3390/genes2040853

**Published:** 2011-11-04

**Authors:** Jekaterina Jutkina, Eeva Heinaru, Eve Vedler, Jaanis Juhanson, Ain Heinaru

**Affiliations:** Chair of Genetics, Institute of Molecular and Cell Biology, University of Tartu, Riia Street 23, Tartu 51010, Estonia; E-Mails: eeva.heinaru@ut.ee (E.H); eve.vedler@ut.ee (E.V.); jaanis.juhanson@ut.ee (J.J.); ain.heinaru@ut.ee (A.H.)

**Keywords:** IncP-9 plasmids, aromatic degraders, Baltic Sea bacteria, *Pseudomonas*

## Abstract

Plasmids are mobile genetic elements that provide their hosts with many beneficial traits including in some cases the ability to degrade different aromatic compounds. To fulfill the knowledge gap regarding catabolic plasmids of the Baltic Sea water, a total of 209 biodegrading bacterial strains were isolated and screened for the presence of these mobile genetic elements. We found that both large and small plasmids are common in the cultivable Baltic Sea bacterioplankton and are particularly prevalent among bacterial genera *Pseudomonas* and *Acinetobacter.* Out of 61 plasmid-containing strains (29% of all isolates), 34 strains were found to carry large plasmids, which could be associated with the biodegradative capabilities of the host bacterial strains. Focusing on the diversity of IncP-9 plasmids, self-transmissible *m*-toluate (TOL) and salicylate (SAL) plasmids were detected. Sequencing the *repA* gene of IncP-9 carrying isolates revealed a high diversity within IncP-9 plasmid family, as well as extended the assumed bacterial host species range of the IncP-9 representatives. This study is the first insight into the genetic pool of the IncP-9 catabolic plasmids in the Baltic Sea bacterioplankton.

## 1. Introduction

Microbial adaptation to different types of pollutants entering the environment is strongly associated with the horizontal gene transfer. Horizontal spread of existing catabolic pathways as well as the evolution of novel ones is mediated by mobile genetic elements, especially by catabolic plasmids [1]. Catabolic plasmids of IncP group in particular have been the subject of interest [2]. The degradation of naturally occurring pollutants is mediated mostly by IncP-2, IncP-7 and IncP-9 catabolic plasmids, but the detoxification of xenobiotics, *i.e.*, man-made chemical compounds is often encoded by IncP-1 representatives [1,3,4]. It has been shown also that the resistance to antibiotics in *Pseudomonas* species is associated with the IncP-9 plasmids [5,6].

Based on divergence in *repA* and *oriV* sequences the IncP-9 plasmids are assigned to nine subgroups (α to ι) and two major clusters defined as pWW0 and pDTG1 branches. Additionally, a few certain types of atypical IncP-9 plasmids are exempted from comparative phylogenetic analyses, revealing high level of sequence diversity among the IncP-9 plasmid family [6]. Wide geographical spread of IncP-9 plasmids appears to be strongly correlated with the existence of strong selective pressure [5].

Despite extensive research on catabolic plasmids worldwide, there is a large knowledge gap regarding the plasmid pool of the Baltic Sea. Since oil and oil spills are considered to be the major threat to Baltic Sea ecosystem because of the large amount of oil used, transported and stored in the region [7], it could be a selective environment for microorganisms carrying catabolic plasmids. It has been shown by Leitet and colleagues [8] that 19% of the 130 different Baltic Sea bacterial isolates contained small plasmids of unknown function with predominant genome size of 2-4 kb. Plasmid-containing bacterial hosts were found to be phylogenetically diverse, belonging to *Alphaproteobacteria* and *Gammaproteobacteria, Actinobacteria* and *Bacteroidetes* phylogenetic groups. A small cryptic plasmid pSFKW33 from bacterial strain *Shewanella* sp. 33B isolated from the Baltic Sea surface water was recently sequenced and characterized [9]. However, to our knowledge, no bacteria carrying large catabolic IncP-9 plasmids have been isolated from this ecosystem.

The aim of the present research was to identify plasmid-containing biodegradative bacterial strains from the Baltic Sea water and to screen bacterial isolates for the presence of IncP family representatives, focusing on the diversity of IncP-9 plasmids.

## 2. Results and Discussion

Although degradative and drug resistant plasmids from the plasmid families IncP-1, IncP-2, IncP-4 (IncQ), IncP-7 and IncP-9 are of obvious significance in different environments [2,5,6,10], little is known about the plasmid pool of marine ecosystems. Only a few studies revealed that 19%–30% of isolated seawater bacterial strains may carry plasmids [8,11,12]. In addition, individual plasmids from seawater samples were also characterized using culture-based and culture-independent approaches [9,13]. Several plasmids of IncP-1 family have been isolated from marine biofilm and thoroughly analyzed [14]. Nevertheless there is still a lack of knowledge about plasmids belonging to incompatibility group P. Therefore we concentrated our research on detection of degradative plasmids and IncP representatives in the Baltic Sea water isolates.

### 2.1. Screening of Plasmid-Containing Isolates for the Presence of IncP Plasmids

Based on the ability to utilize aromatic compounds naphthalene, *m*-toluate, salicylate, phenol and/or benzoate as sole energy and carbon sources, a total of 209 bacterial strains were isolated from four sampling sites for further analyses. These components were chosen because the catabolic pathways for the degradation of these chemicals are often encoded by plasmids [2,3,6,15]. The bacterial strains obtained from nonselective R2A media were taken for further analysis only if they tested positive on mentioned selective media. The BOX-PCR fingerprint patterns of all isolates were generated using primer BOXA1R (Table 1) in order to exclude identical isolates (clone strains) from further analyses.

**Table 1:** PCR primers used in the study.

**Primer target**	**Primers**	**Nucleotide sequence (5′**−**3′)**	**Annealing temp. (°C)**	**Extension time (min)**	**Product size (bp)**	**References**
*repA* of IncP-9 family	rep9F	CGCGGYACWTGGGTWCAGAC	58	1	446	[16]
	rep9R	GGYGGWTCCATRCCWGGRCC				
*repA* of IncP-9 family	IncP9 Fw	CMCARCGCGGYACWTGGG	53	1	400	This study
	IncP9 Rev	GTCGGCAICTGCTTGAGCTT				[17]
*repA* of IncP-7 family	IncP7 Fw	ATCCAAGAAGGCCCATGCCG	59	1	505	This study
	IncP7 Rev	CTCAACTCGTAGCTGACATCC				
*repA* homologue of IncP-1 family	IncPl Fw	CTGCGSGCCGANGAYGACG	57	1	462	This study
	IncP1_Rev	GGYGGAATCCGANCCGCAC				
*repA* of IncQ family	IncQF2	CTRCARCTGGCCGCACAG	55	1	494	This study
	IncQR2	AGGTAGGACTGCCAGCGG				
IncQ family	IncQ oriV 1	CTCCCGTACTAACTGTCACG	57	1	436	[18]
	IncQ oriV 2	ATCGACCGAGACAGGCCCTGC				
16S rRNA gene	PCRI	AGAGTTTGATCATGGCTCAG	53	2	∼1.5 kb	[19]
	PCRII	TACGGTTACCTTGTTACGACTT				
	785FL	GGACTACGGATTAGATACCCTGGTAGTCCI	63	0,5	156	[20]
	919R	CTTGTGCGGGTCCCCGTCAAT				
Repeated regions in chromosome	BOXA1R	CTACGGCAAGGCGACGCTGACG	53→68	1→8	Various	[21]

Based on the electrophoretic mobility of the DNA profiles of bacterial isolates, 61 bacterial strains (29% out of the 209 isolates) were found to carry single or multiple plasmids, and 34 plasmid-containing strains carried large plasmid(s) (Table 2). Each DNA band of a strain's electrophoregram located above or below chromosomal DNA band was defined as individual large or small plasmid, respectively. However, multiple bands could also be different forms of the same plasmid, which remains to be elucidated in future studies. The high proportion of the large plasmid-bearing strains, compared with the prevalence of small plasmids found in other studies mentioned above, could be influenced by the biodegradative capabilities of the isolates, and with the potential presence of catabolic plasmids. The majority of the strains (53) were found to degrade benzoate, 44 isolates degraded phenol, 34 degraded *m*-toluate, 20 degraded naphthalene, and 21 isolates degraded salicylate (Table 2). The catabolic capacities of plasmid-containing isolates as well as phylogenetic analysis of 16S rRNA gene sequences are shown in Table 3.

**Table 2:** Catabolic properties of plasmid-containing bacterial strains isolated from the Baltic Sea surface water samples A, B, C, D and their phenotypes.

**Strain**	**The most similar homologue from the GenBank database, acc. nr.**	**Identity**	**No of plasmid DNA bands** *	**Ability to degrade aromatic compounds**				
				**Phe**	**Benz**	**Tol**	**Sal**	**Nah**
*Gammaproteobacteria*									
AB1	*Acinetobacter tjernbergiae*, FR822986	99%	1L	+	+	+	-	+
AP3	*Aeromonas rivuli*, FJ976900	100%	1L + 4S	+	+	-	-	+
A2	*Acinetobacter* sp., FJ192980	99%	1S	+	+	+	-	-
A8	*Pseudomonas putida*, AM411059	99%	1L	+	+	-	+	-
A12	*Acinetobacter haemolyticus*, AY047216	99%	3S	+	+	+	-	-
A17	*Pseudomonas fluorescens*, GU198122	99%	1L	-	+	-	+	-
A19	*Acinetobacter calcoaceticus*, AY800383	99%	1L	+	-	+	-	-
A30	*Acinetobacter* sp., FJ192980	99%	2S	+	+	+	-	-
A34	*Acinetobacter calcoaceticus*, AY800383	99%	1L	+	+	+	+	-
A52	*Acinetobacter haemolyticus*, AY047216	99%	1S	+	+	+	-	-
A56	*Acinetobacter calcoaceticus*, AY800383	99%	1L	-	-	-	+	-
A67	*Acinetobacter* sp., FJ192980	99%	2S	+	+	+	-	-
A71	*Acinetobacter* sp., FJ192980	99%	3S	+	+	+	-	-
2Aphe4	*Pseudomonas stutzeri*, FJ859914	100%	1L	+	-	-	-	-
2A7	*Pseudomonas stutzeri*, FJ859914	100%	1L	+	-	-	-	-
2A20	*Pseudomonas stutzeri*, EU652047	99%	1L	+	+	+	-	-
2A54 **	*Pseudomonas stutzeri*, EU652047	100%	1L + 1S	+	+	+	-	-
B10v	*Pseudomonas stutzeri*, JF727659	99%	4S	-	-	+	-	-
B17	*Pseudomonas putida*, GU186116	99%	1L + 1S	+	+	+	-	+
B37	*Pseudomonas putida*, GU186116	99%	1S	+	+	-	-	+
B43	*Acinetobacter johnsonii*,DQ911549	99%	6S	+	+	+	-	+
2Bsal	*Pseudomonas migulae*, EU111725	99%	1S	+	+	-	+	+
2B31	*Pseudomonas veronii*, AB494445	99%	1S	-	+	+	-	+
2B49	*Pseudomonas guinae*, AM491811	99%	3S	-	-	+	-	-
C14 **	*Pseudomonas putida*, DQ178233	99%	2L + 3S	+	+	-	+	-
C70	*Pseudomonas pseudoalcaligenes*, NR 037000	99%	1L	+	+	-	+	+
CN1b	*Acinetobacter junii*, AM184300	100%	2S	+	+	+	+	+
CN3b	*Acinetobacter junii*, AM184300	100%	1S	+	+	+	-	+
CS2	*Acinetobacter junii*, AM184300	100%	1L	+	+	-	-	-
CP1	*Acinetobacter junii*, AM184300	100%	1L + 1S	+	+	+	-	+
2C20v	*Pseudomonas stutzeri*, AJ244724	99%	1L	-	+	-	-	-
2C31 **	*Pseudomonas putida*, AF094746	100%	1L	-	+	+	+	-
2C43 **	*Pseudomonas putida*, AF094746	100%	1L	+	+	-	+	-
2C48	*Acinetobacter johnsonii*, DQ911549	99%	5S	-	+	-	-	-
2C52	*Acinetobacter johnsonii*, DQ911549	99%	5S	+	+	+	-	+
2C56	*Acinetobacter johnsonii*, EU337121	99%	3S	+	+	+	-	-
2C68	*Pseudomonas putida*, AF094746	100%	1L	-	+	-	-	-
2Cphe2	*Acinetobacter johnsonii*, DQ911549	99%	5S	+	+	+	-	+
2Ctol2 **	*Pseudomonas putida*, AF094746	100%	1L	+	+	+	+	+
2Ctol3	*Pseudomonas stutzeri*, FJ859914	99%	1L	+	-	-	-	-
D2RT	*Pseudomonas migulae*, EU111725	99%	1L	-	+	+	+	-
D10	*Pseudomonas aeruginosa*, FJ620575	99%	1L	-	+	-	+	-
D14	*Acinetobacter johnsonii*, DQ911549	98%	4L + 6S	+	+	-	-	-
D19	*Acinetobacter johnsonii*, EU337121	99%	5S	+	+	-	-	-
D25	*Pseudomonas lini*, AY035996	99%	1L + 1S	-	+	-	+	-
D65lp	*Pseudomonas anguilliseptica*, AF439803	99%	1S	-	+	-	-	-
D65v	*Acinetobacter schindleri*, GU339299	99%	1S	+	+	-	-	+
D67 **	*Pseudomonas migulae*, EU111725	99%	1L	-	+	+	+	-
D69v	*Pseudomonas lini*, AY035996	99%	2S	+	+	+	+	+
DN1	*Acinetobacter lwoffii*, FJ860882	99%	1L + 3S	+	+	-	-	+
DP1	*Acinetobacter lwoffii*, FJ860882	99%	3S	+	+	-	-	+
DR2A1	*Acinetobacter lwoffii*, FJ860882	99%	2S	+	+	+	-	+
2Dphe2	*Pseudomonas stutzeri*, AJ244724	100%	2L	+	+	-	-	-
2D47 **	*Pseudomonas stutzeri*, EU652047	100%	2S	+	+	+	+	-
2D49 **	*Pseudomonas stutzeri*, EU652047	100%	2S	+	+	+	+	-
2D61 **	*Pseudomonas putida*, CP002290	100%	1L	+	+	+	+	-
2D67	*Acinetobacter johnsonii*, EU337121	99%	1S	+	+	+	-	+
2D68 **	*Pseudomonas putida*, CP002290	100%	1L	+	+	-	+	-
*Betaproteobacteria*									
2A10	*Acidovorax radicis*, HM027578	99%	1L	-	+	-	-	-
*Alphaproteobacteria*									
D69k	*Novosphingobium* sp., AB360760	100%	1L	-	+	+	+	-
2D23	*Sphingomonas xenophaga*, AY611716	99%	2L + 1S	-	-	+	-	-

* Plasmids were separated according to size by agarose gel electrophoresis. Each band of plasmid DNA located above or below chromosomal DNA band was defined as individual large plasmid (L) or small plasmid (S), respectively; + denotes positive growth on phenol (Phe), benzoate (Benz), *m*-toluate (Tol), salicylate (Sal), naphthalene (Nah);

** IncP-9 positive isolates.

**Table 3:** Transferability of IncP-9 plasmids in mating experiments with *P. putida* PaW340.

**Strain**	**Identified species**	**Sampling point**	Transconjugant and its phenotype *	Transfer frequency **
**2A54**	*P. stutzeri*	A	No transfer	
**C14**	*P. putida*	C	No transfer	
**2C31**	*P. putida*	C	No transfer	
**2C43**	*P. putida*	C	PaW340 (p2C43), Sal^+^, Sm^r^, Trp^−^	<1.0 × 10^−8^
**2Ctol2**	*P. putida*	C	PaW340 (p2Ctol2), Sal+, Sm^r^, Trp^−^	3.89 (±0.05) × 10^−6^
**D67**	*P. migulae*	D	PaW340 (pD67), Tol^+^, Sm^r^, Trp^−^	2.12 (±0.53) × 10^−4^
**2D47**	*P. stutzeri*	D	No transfer	
**2D49**	*P. stutzeri*	D	No transfer	
**2D61**	*P. putida*	D	PaW340 (p2D61), Tol^+^, Sm^r^, Trp^−^	4.37 (±0.11) × 10^−5^
**2D68**	*P. putida*	D	PaW340 (p2D68), Sal^+^, Sm^r^, Trp^−^	1.14 (±0.31) × 10^−4^

* Sal^+^, Tol^+^, the ability to degrade salicylate and m-toluate, respectively; Sm^r^, resistance to streptomycin, Trp^−^, requirement for tryptophan in growth medium;

** Transconjugants per donor cells.

According to the sequence comparison of amplified 16S rRNA genes (primers shown in Table 1), all 61 plasmid-containing bacterial strains belonged to the phylum *Proteobacteria*. The majority of them belonged to the genera *Pseudomonas* (31) and *Acinetobacter* (26) (Table 2). The strong dominance of *Gammaproteobacteria* representatives (95%) was supported additionally with the strain AP3, which was affiliated to the genus *Aeromonas*. Class *Betaproteobacteria* was represented only by a single bacterial strain 2A10 of genus *Acidovorax*. Class *Alphaproteobacteria* was represented by two isolates D69k and 2D23 belonging to the genus *Novosphingobium* and *Sphingomonas*, respectively.

All plasmid-containing bacterial strains were screened for the presence of IncP family plasmids by using primers listed in Table 1. It is known so far that among transmissible plasmids the IncP-1 representatives have broader host range compared to plasmids of IncP-7 and IncP-9 families [2,22]. Non-self-transmissible plasmids from the IncQ (IncP-4) family are mostly small plasmids with broad host range, which could be mobilized by conjugative plasmids of different groups [23]. In this study, neither IncP-1, IncP-7, nor IncQ plasmids were detected. However, 10 IncP-9 positive bacterial strains were detected, which all belonged to the genus *Pseudomonas*. Five strains (2D47, 2D49, 2D61, 2D68 and D67) were isolated from sampling point D, 4 strains (C14, 2C31, 2C43 and 2Ctol2) represented sampling point C and the single isolate 2A54 was obtained from sampling point A. Sampling points A, C and D were located in regions with increased human activities, which may provide selective components for proliferation of IncP-9 family plasmids. Contrarily, the absence of isolates carrying IncP-9 plasmids from sampling point B (water sample taken from off-shore region in the Gulf of Finland) may refer to lower level of potential biodegradative selective markers in this area.

### 2.2. Analysis of Pseudomonas Strains Bearing IncP-9 Plasmids and Mating Experiments

Although the 16S rRNA gene is the basis of current bacterial taxonomy, it is often insufficient to distinguish closely related bacterial species [24]. In order to analyze 10 *Pseudomonas* strains bearing IncP-9 plasmids in more detail, 16S rRNA gene analyses were supported by additional morphological, physiological and biochemical data. To investigate the transferability of plasmids of IncP-9 positive isolates, conjugal mating experiments with *P. putida* PaW340 as a recipient were performed (Table 3).

Three IncP-9 positive strains (2A54, 2D47 and 2D49) belong to the species *P. stutzeri* (Table 2). *P. stutzeri* strains have been frequently isolated from water environments, including marine water and sediments as well as wastewater samples, representing large metabolic versatility of isolates. Ability of several *P. stutzeri* strains to degrade naphthalene and resistance to mercury were shown to be encoded by plasmids [25,26]. *P. stutzeri* has been a model organism for the study of natural transformation and horizontal gene transfer [27]. However no evidence so far has been found for the presence of IncP-9 plasmids in natural isolates of this bacterial species. We were also not able to determine the affiliation of amplified IncP-9 plasmid family *repA* sequences of plasmids in *P. stuzeri* strains 2A54, 2D47 and 2D49. Only strain 2A54 carried one large plasmid DNA, while the other two (2D47 and 2D49) carried several small plasmids (Table 2). Although we could not detect plasmid transfer in *P. putida* PaW340, the ability of plasmids to self-transfer should not be excluded (Table 3). Thus the determination of the IncP-9 plasmid location as well as relatedness to catabolic features in *P. stutzeri* strains and transferability of plasmids remains for future studies.

*Pseudomonas migulae* strain D67 contains self-transmissible TOL plasmid of IncP-9 family (Table 3). *Pseudomonas migulae* strains have been isolated form water and soil samples [28,29] and their ability to harbor pWW0 TOL plasmid was demonstrated also previously in microcosms with oil-contaminated soil [28].

The majority of bacterial host strains carrying IncP-9 plasmids were affiliated to *Pseudomonas putida* species (6 out of 10). All *P. putida* strains contained large plasmids (Table 2). Four self-transmissible plasmids were identified—3 SAL plasmids (in strains 2C43, 2Ctol2 and 2D68) and 1 TOL plasmid (in strain 2D61). Plasmid transfer to *P. putida* PaW340 was not detected for *P. putida* strains C14 and 2C31 (Table 3). Additional analysis should be carried out to verify the transferability of the plasmids pC14 and p2C31.

Our previous study demonstrated horizontal gene transfer of large NAH IncP-9 plasmid pNAH20 under natural conditions in soil [16]. We assume that IncP-9 large conjugative biodegradative plasmids could be excellent candidates for horizontal gene transfer in the Baltic Sea as well. So far, large catabolic plasmids of IncP-9 family have been isolated only from the genus *Pseudomonas,* indicating possible narrow host range of this plasmid group. The bacterial host range of IncP-9 plasmids determined by us is also restricted only to the genus *Pseudomonas*. Although the genus *Pseudomonas* is one of the most ubiquitous bacterial genera known so far [24], IncP-9 plasmids have been isolated only from a few species—*P. putida, P. fluorescens, P. aeruginosa* and *P. aureofaciens* [3,6]. Our study extended the bacterial host species range of the IncP-9 family, revealing IncP-9 plasmids in *P. stutzeri* and *P. migulae* as well.

### 2.3. Diversity of the Baltic Sea IncP-9 Plasmids

**Figure 1:**
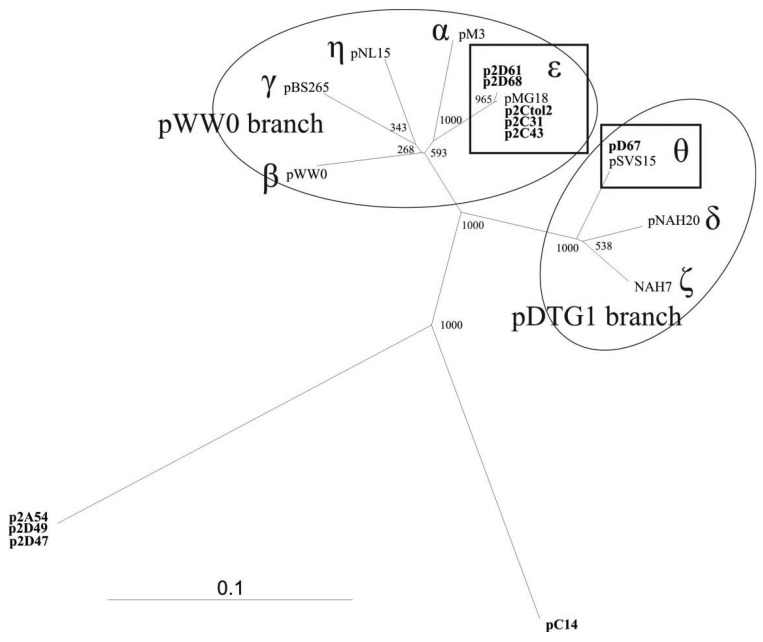
Phylogenetic analysis of *repA* gene sequences amplified with IncP-9 plasmid-targeted primers. Neighbor-joining unrooted phylogenetic tree of IncP-9 plasmid family is constructed based on 355 bp partial *repA* gene sequence analysis. The plasmids isolated in this study are shown in bold letters. Ovals define plasmid branches: pWW0-branch and pDTG1-branch. Plasmid subgroups of particular interest are rectangle shaped. Bootstrap values (out of 1,000) are shown adjacent to branch nodes. The scale bar shows the number of substitutions per site. pNL15, pBS265, NAH7, pM3, pWW0, pMG18, pSVS15, pNAH20 sequences represent the known different IncP-9 subgroups, named with the letters of Greek alphabet.

To study the diversity of the IncP-9 plasmids isolated from the Baltic Sea water samples, nucleotide sequencing of the 446 bp *repA* gene fragment was performed (primers rep9F/rep9R shown in Table 1). Sequences were aligned against reference plasmid sequences obtained from GenBank as well as against each other. The results demonstrated significant diversity of these plasmids. Phylogenetic analyses revealed that majority of plasmids belonged to two known subgroups of IncP-9 family while several plasmids represented new phylogenetic lineages within this family (Figure 1).

The five IncP-9 *repA* sequences of the host strain *P. putida* (p2Ctol2, p2C43, p2C31, p2D61, p2D68) represented ε subgroup (Figure 1). DNA sequence identity within ε subgroup was 99%–100%, thereby p2Ctol2, p2C43, p2C31 showed 100% identity to antibiotic resistance plasmids pMG18 and R2. A single *repA* sequence of the plasmid pD67 of *P. migulae* was affiliated to subgroup θ. Sequence alignment revealed 100% identity between *repA* fragments of pD67 and pSVS15. Both plasmids pD67 and pSVS15 carry genes for degradation of toluene [30].

The three IncP-9 plasmids of *P. stutzeri* (p2A54, p2D47, p2D49) formed a new phylogenetic lineage showing 54–57% DNA identity with the other subgroups. BLASTN search analysis revealed 75% identity between *repA* sequences of p2A54, p2D47, p2D49 and pACMV2. The plasmid pACMV2 (NC_015187) is ∼65 kbp large, it is originated from the bacterial strain *Acidiphilium multivorum* AIU301, and exhibits resistance to arsenate and arsenite [31].

Comparative analysis of the plasmid pC14 originated from isolate P. putida C14 revealed, that repA of this plasmid has only 56–64% DNA similarity with sequences from different subgroups and therefore forms another separate lineage in the phylogenetic tree of IncP-9 plasmids (Figure 1).

Amplification of new phylogenetically distant plasmids extended the knowledge about diversity of IncP-9 family. The presence of atypical plasmids classified only by incompatibility testing was already mentioned by Sevastsyanovich [6] referring to unascertained diversity of IncP-9 plasmids. Our research confirmed data on large diversity of IncP-9 family summarized by Sevastsyanovich [6] and enclosed additional lineages to previously assumed two major clusters pDTG1 and pWW0.

### 2.4. Occurrence of IncP-9 Plasmids in Extracted Total Community DNA

Real-time PCR analysis was carried out in order to estimate the presence of IncP-9 plasmids in different sampling sites A, B, C and D (Table 4).

**Table 4:** Abundance of IncP-9 *repA* gene and 16S rRNA gene and the log10 transformed ratio of IncP-9 to 16S rRNA gene copies in different sampling sites.

**Sample site**	IncP-9 copy number/L seawater *	16S rRNA gene copy number/L of seawater *	Log10 (IncP-9/16S rRNA) *
B (Gulf of Finland)	5.76 (±0.36) × 10^5^	1.69 (±0.14) × 10^9^	−3.45 (±0.03)
A (Tallinn)	1.47 (±0.14) × 10^6^	3.09 (±0.23) × 10^9^	−3.32 (±0.04)
C (Narva)	3.34 (±0.37) × 10^5^	3.11 (±0.30) × 10^9^	−3.97 (±0.05)
D (Pärnu)	2.42 (±0.49) × 10^6^	6.07 (±0.37) × 10^9^	−3.40 (±0.08)

* Means and standard deviations (in parentheses) are presented.

Contrary to culture-based approach, real-time PCR analysis of environmental samples revealed the presence of the IncP-9 *repA* gene sequences in all four sampling sites. IncP-9 *repA* gene copy numbers in the water samples were between 3.34 × 10^5^ and 2.42 × 10^6^, while 16S rRNA gene copy numbers were in the range of 1.69−6.07 × 10^9^ copies/L of seawater. The log10 transformed ratio of IncP-9 *repA* gene to 16S rRNA gene copies varied from −3.32 to −3.97. The lowest ratio (−3.97) was observed in sampling point C (Table 4). Although PCR data is not comparable to the results obtained from culture-based approach, both analyses complement each other and provide better insight into the distribution of IncP-9 plasmids in the Baltic Sea ecosystem.

## 3. Experimental Section

### 3.1. Sampling Sites and Collection of Samples

**Figure 2:**
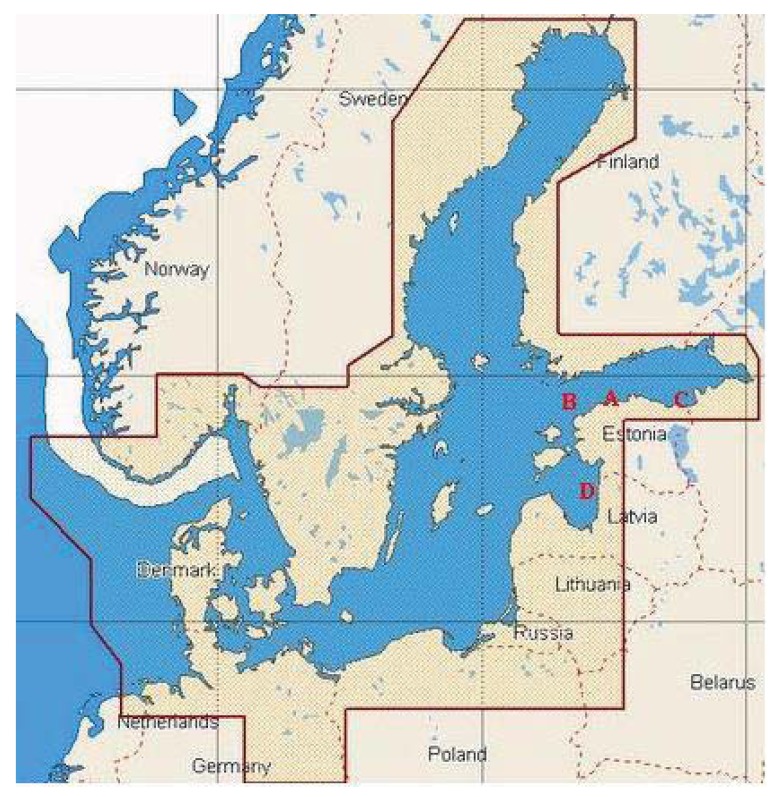
Water samples collection sites from the Baltic Sea regions A, B, C, D near Estonia. Map of the Baltic Sea was obtained from Internet source [32] and modified.

The surface water samples were collected in August and September 2008 and 2009 along the Baltic Sea shoreline in depth of approximately 1 m using sterile 12 L canisters. The water samples were stored in sterilized glass bottles at 4 °C until analysis. A total of 8 samples were collected near Tallinn (59°32′12″ N, 24°41′18″ E), Narva (59°28′30″ N, 28°00′30″ E) and Pärnu (57°37′00″ N, 23°37′00″ E), as well as from offshore water in the Gulf of Finland (59°28′60″ N, 22°57′00″ E) (Figure 2). Sampling sites were named with capital letters A (Tallinn), B (the Gulf of Finland), C (Narva) and D (Pärnu), and distances of the sampling sites from the shoreline were ca 10 km, 50 km, 2 km, and 50 km, respectively. In addition, sampling sites C and A are located in the near proximity to the oil shale mining and processing area (ca 2 km), and harbor terminals (ca 10 km), respectively. Therefore we assume that these sampling sites are heavily influenced by human activities, exposed to oil in particular. Sampling point B is located more than 100 km from the closest towns Tallinn and Helsinki, sampling point D is located ca 85 km from the closest towns Pärnu and Riga.

### 3.2. Isolation of Cultivable Aromatic Compounds-Degrading Bacteria from Seawater

Five structurally simple, readily degradable aromatic compounds, phenol (Phe), benzoate (Benz), m-toluate (Tol), salicylate (Sal) and naphthalene (Nah) were used as model substrates for detection of catabolic plasmids. Two approaches were applied to isolate aromatic compounds-degrading bacteria from seawater: direct selection and enrichment. Direct selection involved plating of seawater on low-nutrient solid R2A [33] and minimal medium plates containing M9 salts [34] and trace elements [35] supplemented with 2.5 mM Phe, Benz, Tol or Sal and in vapor phase Nah as the sole sources of carbon. The enrichment approach involved three serial enrichments, in which the first was done into the seawater, the second and third in M9 media, were performed in 250-mL sterile flasks at 15 °C on rotary shaker (130 r.p.m.). Phe, Benz, Tol and Sal were added to 50 mL of liquid media at a final concentration of 2.5 mM and Nah 0.1% w/v. Bacterial strains from different enrichments were isolated after serial dilution and plating on R2A agar and selective media containing corresponding carbon sources during 10 days. Morphologically different colonies were picked from the plates, purified and studied with BOX-PCR for elimination of siblings [15]. Pure bacterial cultures were stored in 20% glycerol at −80 °C.

### 3.3. Extraction of Total Community DNA

Two litres of collected water samples from different sampling sites were run through a sterile filter with pore size of 0.2 μm (Sartorius AG) and stored at −80 °C. Filters were used for extraction of total community DNA using PowerSoil™ DNA Isolation Kit (Mo Bio Laboratories).

### 3.4. Real-Time PCR

The qPCR was performed in the real-time PCR system Rotor-Gene ^®^ Q (Qiagen) with a total reaction volume of 10 μL. Reaction mixture included 1× Maxima SYBR Green Master Mix (Fermentas), both primers (either IncP9_Fw and IncP9_Rev, or 785FL and 919R; Table 1) at concentration of 0.4 μM and 1 μL of extracted total community DNA. The reaction conditions were: 50 °C for 2 min, 95 °C for 10 min, followed by 40 cycles of denaturation at 95 °C for 15 s, annealing at 53 °C (63 °C for 16S rRNA gene) for 30 s, and extension at 72 °C for 30 s. The fluorescence intensity of SYBR Green was measured automatically at the end of the extension step. At the end of run, melting curve analyses was performed ramping the temperature from 82 °C to 95 °C, using 3 s and 0.35 °C interval with continuous fluorescence recording. Data from the qPCR were analyzed with Rotor-Gene Series software, version 2.0.2.4 (Qiagen). qPCR was performed in triplicate for each sample, including samples for standard curve and negative control.

For standard curves, conventional PCR with the same primers (IncP9_Fw and IncP9_Rev, 785FL and 919R; Table 1) was performed to amplify either the target IncP-9 *repA* fragment sequence or 16S rRNA gene sequence from the positive control strain *Pseudomonas fluorescens* PC20. This strain carries IncP-9 plasmid pNAH20 completely sequenced and characterised in our previous work [16] (Table 5). Amplified products was run on 1% (wt/vol) agarose gel followed by extraction from the gel using QIAquick Gel Extraction Kit (Qiagen) according to the protocol provided by manufacturer. The concentration of the purified DNA fragments was determined with spectrophotometer Nanodrop ND-1000 (Nanodrop Technologies Inc, USA). Standard curve for the qPCR was obtained using tenfold serial dilutions of the DNA fragments as a template in qPCR reaction. Copy numbers were calculated from the standard curves assuming that the average molecular mass of a double stranded DNA molecule is 660 g mol^−1^. Highly linear standard curves (R^2^ values > 0.99; PCR efficiency = 99%) over the dilution range of 10^8^−10^3^ copies were obtained for both, IncP-9 *repA* gene and 16S rRNA gene sequences. The initial target gene copy numbers in seawater samples were deduced from the standard curves.

**Table 5:** Reference bacterial strains used in this study.

**Bacterial strain**	**Phenotype**	**Source of reference**
*Pseudomonas fluorescens* PC20	Wild-type strain harboring IncP-9 plasmid pNAH20, Phe^+^, Sal^+^, Nah^+^	[16]
*P. fluorescens* PC24	Wild-type strain harboring IncP-7 plasmid pPHE24	[15]
*Achromobacter xylosoxidans* subsp. *denitrificans* EST4002	Wild-type strain harboring IncP1 plasmid pEST4011	[36]
*P. putida* PaW85(pEST1412)	PaW85 derivative harboring IncQ plasmid pEST1412	[37]

### 3.5. General DNA Manipulations

Bacterial genomic DNA from isolates was prepared using UltraClean microbial DNA isolation kit (Mo Bio Laboratories) according to the manufacturer's instructions. Total DNA was used as a template for the amplification of 16S rRNA and *repA* gene fragments [38]. PCR was performed with *Taq* polymerase (MBI Fermentas), the primers used together with annealing temperatures and extension times are listed in Table 1. All other PCR conditions have been described previously [16,39].

Bacterial isolates growing on aromatic compounds were screened for the presence of plasmids by the procedures of Connors and Barnsley [40]. In brief, bacterial biomass was grown on solid aromatic medium and harvested from agar surface, plasmid DNA was extracted from this biomass by mild alkaline lysis followed by DNA precipitation, the dissolved DNA was immediately analyzed by electrophoresis on 0.8% (wt/vol) agarose gel. Each DNA band in UV visualized agarose gel, which located above or below chromosomal DNA band was defined as individual large or small plasmid, respectively (Table 2).

Conjugal mating experiments conducted in this study were described in our previous work [16]. The recipient strain was *P. putida* PaW340 (Phe^−^, Nah^−^, Sal^−^, Tol^−^, Sm^r^, Trp^−^) [41]. Transconjugants were selected on M9 mineral medium agar plates containing tryptophan (40 g L^−1^) and streptomycin (1 g L^−1^) and either Phe, Tol, Sal (2.5 mM) or Nah (in vapours phase). BOX-PCR was used to confirm the genetic background of transconjugants. The presence of plasmids in transconjugants was verified by amplification of *repA* gene fragment by PCR as previously described [16].

The reference strains used as controls for IncQ, IncP-1, IncP-7 and IncP-9 specific primers are listed in Table 5.

### 3.6. Primer Design

The primers IncP7_Fw and IncP7_Rev were designed to completely match the conserved regions in *repA* genes of three IncP7 plasmids—pCAR1, pND6-1 and pWW53. The primers for IncP-1 *repA* homologue are based on alignment of the representatives of four of the five subgroups of this plasmid family—RK2, R751, pADP-1, pQKH54 and pEST4011. As this plasmid family is more diverse, 2 and 3 mismatches were allowed in case of IncP1_Fw and IncP1_Rev, respectively. All these mismatches are in 5′-halves of the primers. The primers IncQF2 and IncQR2 were designed based on the IncQ plasmids pMS260, pCHE-A, pCCK1900, pND1, pIE1115, RSF1010 and pIE1130 by using conservative regions of the *repA* gene sequences. No mismatches were allowed.

Two primer sets were designed by us to target the *repA* gene sequences of the IncP-9 plasmid group. The primers rep9F and rep9R were designed according to the aligned sequences of the *repA* gene sequences of the IncP-9 plasmids pWW0, pM3, NAH7 and pDTG1, belonging to different subgroups of this family; no mismatches were allowed [16]. For qPCR analysis IncP9_Fw/ IncP9_Rev primer set was chosen because of a smaller size (400 bp) of amplified PCR product (446 bp for rep9F/rep9R primers). These primers were designed by Greated and colleagues [17], but modified by us by adding 3 redundant positions to the primer IncP9_Fw to broaden its binding range. These modifications were based on the alignment of the same abovementioned IncP-9 plasmids. The specificity of the both IncP-9 primer sets was tested using standard PCR amplification performed on IncP-9 plasmid containing cultured bacterial strains as well as using the total community DNA extracted from the environmental samples A, B, C and D. PCR products yielded by amplification of *repA* gene from environmental samples underwent cloning and sequencing. At least 5 clones from each library were randomly picked and analyzed. Totally 36 and 20 sequences originating from the PCR amplifications with IncP9_Fw/IncP9_Rev and rep9F/rep9R primer sets, respectively, were submitted to BLAST search. BLAST analysis revealed the presence of *repA* gene sequences belonging to two known branches of IncP-9 family—pWW0 and pDTG1. PCR products obtained from cultured bacterial strains were sequenced directly. The results indicate that the both primer sets are suitable for detection of IncP-9 plasmids.

### 3.7. Sequencing and Phylogenetic Analysis

Nucleotide sequencing was carried out on a 3730*xl* DNA Analyzer (Applied Biosystems) using the BigDye ^®^ Terminator v3.1 Cycle Sequencing Kit (Applied Biosystems) according to the manufacturer's protocol. Multiple alignments were done using neighbor-joining method by CLUSTALX program. Phylogenetic trees were visualised with TreeView. Sequence comparisons were done using BLAST from NCBI. For phylogenetic analysis of IncP-9 plasmids the following sequences were obtained from database: pM3 AF078924, pWW0 NC_003350, pMG18 EU499632, pSVS15 EU499641, pNAH20 NC_012674, pNL15 EU499658, NAH7 AB237655, pBS265 EU499653.

### 3.8. Nucleotide Sequence Accession Numbers

The nucleotide sequences obtained in this study have been assigned the GenBank accession numbers from JN228272 to JN228332 for 16S rDNAs, and from JN228333 to JN228342 for *repA* sequences.

## 4. Conclusions

In the present study we isolated 209 bacterial strains from the Baltic Sea water able to degrade different aromatic compounds. Among them we found 61 strains containing one or several plasmids, all these strains were screened for the presence of IncP plasmids. The majority (94%) of the plasmid-bearing isolates belonged to the two genera of *Gammaproteobacteria—Pseudomonas* and *Acinetobacter.* Ten bacterial strains representing different *Pseudomonas* species (P. *putida, P. migulae, P. stutzeri*) were found to carry IncP-9 plasmids. Phylogenetic analysis of the *repA* gene sequences of these plasmids revealed a high diversity, resulting in two new lineages within this plasmid group. Self-transmissible TOL and SAL plasmids were detected in conjugal mating experiments. Conducted qPCR analyses revealed the presence of the IncP-9 *repA* gene sequences in all four sampling sites. Our research is the first insight into the genetic pool of the IncP-9 plasmids in the Baltic Sea bacterioplankton community.
